# Source Camera Identification Techniques: A Survey

**DOI:** 10.3390/jimaging10020031

**Published:** 2024-01-25

**Authors:** Chijioke Emeka Nwokeji, Akbar Sheikh-Akbari, Anatoliy Gorbenko, Iosif Mporas

**Affiliations:** 1 School of Built Environment, Engineering, and Computing, Leeds Beckett University, Leeds LS6 3QR, UK; c.nwokeji2723@student.leedsbeckett.ac.uk (C.E.N.); a.gorbenko@leedsbeckett.ac.uk (A.G.); 2 School of Physics, Engineering & Computer Science, University of Hertfordshire, Hertfordshire AL10 9AB, UK

**Keywords:** source camera identification, camera brand source identification, camera model source identification, sensor pattern noise, image lens optical distortion, camera colour filter array

## Abstract

The successful investigation and prosecution of significant crimes, including child pornography, insurance fraud, movie piracy, traffic monitoring, and scientific fraud, hinge largely on the availability of solid evidence to establish the case beyond any reasonable doubt. When dealing with digital images/videos as evidence in such investigations, there is a critical need to conclusively prove the source camera/device of the questioned image. Extensive research has been conducted in the past decade to address this requirement, resulting in various methods categorized into brand, model, or individual image source camera identification techniques. This paper presents a survey of all those existing methods found in the literature. It thoroughly examines the efficacy of these existing techniques for identifying the source camera of images, utilizing both intrinsic hardware artifacts such as sensor pattern noise and lens optical distortion, and software artifacts like color filter array and auto white balancing. The investigation aims to discern the strengths and weaknesses of these techniques. The paper provides publicly available benchmark image datasets and assessment criteria used to measure the performance of those different methods, facilitating a comprehensive comparison of existing approaches. In conclusion, the paper outlines directions for future research in the field of source camera identification.

## 1. Introduction

The last few years have seen a significant increase in research interest in the field of digital image forensics because the easy availability of advanced and affordable devices has made the acquisition and manipulation of digital media images which used to be a professional job very easily accessible to the public, giving room for untrusted media images and videos being in circulation. According to Su, Zhang, and Ji in [1], the advancement in digital technology and the increasing number of images and video-sharing websites like YouTube, Facebook, Twitter, and other social media platforms have helped the spread of various kinds of less trusted images from individual sources on the internet. Digital forensic investigation is, therefore, more complex nowadays than ever due to this rapid advancement in digital devices and more reliance on it for virtually all human activities with users leveraging the technologies of digital devices that serve both good and malicious purposes and intents. Distinct from other forensic evidence, image and video recording provide a real-time eyewitness account that investigators, prosecutors, and the jury can listen to or see exactly what transpired. It is crucial to acquire a technology capable of proficiently identifying digital devices responsible for capturing images. This capability is essential for supporting law enforcement officers and prosecutors in criminal investigations, covering areas such as child pornography, insurance claims, movie piracy, traffic monitoring, and financial fraud. The challenge then becomes, can it genuinely identify the digital image that came from the alleged camera? The necessity to tackle these and other challenges gave rise to what is today known as image forensics. Identifying the source of an image is a vital aspect of digital forensics, as highlighted by Chen et al. [2], who emphasized that determining the acquisition device of an image as evidence for presentation in court is as crucial as the digital image itself. The goal of addressing the source camera identification problem involves discerning whether a given image was captured with a specific camera, including details about the camera model/brand and the imaging mechanism employed (such as camera, scanner, computer graphics, or smartphone). According to the observations of Thai, Retraint, and Cogranne [3], current active approaches like digital signatures and digital watermarking have drawbacks, as they require the incorporation of specialized information during image generation. Elaborating on these limitations, Chio, Lam, and Wong [4] argued that many images from cameras contain an exchangeable image file format (EXIF) header, which includes information such as the type of digital camera, exposure, date, and time. This information, however, could be maliciously altered and could be destroyed during the process of an image being edited. The drawback of active techniques to source camera identification gave rise to passive techniques which Thai et al. [3] argued have received significant attention in the last decade because they do not impose any constraints and do not require any prior knowledge. Only the suspicious digital image is available to forensic analysts, who can extract meaningful digital information from it to gather forensic evidence, track down the capture device, or discover any alteration therein. According to Bernacki [5], the internal traces or unique artifacts left by the digital camera in each digital image serve as camera fingerprints that are used in passive techniques, and investigating the image acquisition pipeline can offer these internal traces. In this paper a through critical literature survey on existing image camera source identification methods, their assessment criteria, and the publicly available dataset used to assess their performance are presented. Moreover, their performance is compared with each other. This work is an extension of our previous survey published on Nwokeji et al. [6]. The rest of the paper is organized as follows. Section 2 gives an overview of the structure and processing stages of a typical digital camera. Source image camera identification techniques are reviewed in Section 3 and Section 4 presents the conclusion and future directions.

## 2. Digital Camera Image Acquisition and Processing Pipeline

Light travels through a series of filters after entering the camera through the lenses. An infrared filter is an absorptive or reflective filter that blocks infrared radiation that could reduce the sharpness of the created image while only allowing the visible portion of the spectrum to pass. An anti-aliasing filter is used to minimizes aliasing artefacts, a condition in which the finer spatial frequency of the target objects, as opined by Van Lanh et al. [7] such as decorative patterns, cannot be supported by the pixel spacing in the sensor. The image sensor is the heart of every digital camera. A photodiode element known as a pixel is arranged in rows and columns on an image sensor. Each pixel in the pixel array produces an analogue signal proportionate to the amount of light it receives, which according to Fossum [8] is subsequently transformed into a digital signal by digital image processing (DIP) and then it is processed. Charge-coupled device (CCD) image sensors are mostly used by most digital cameras although the complementary metal oxide semiconductor (CMOS) is a popular alternative. Sensor pixels only record light intensity, creating a monochromatic output because they are not colour-sensitive. A colour-filter array (CFA) is placed in front of the sensor to capture the light intensity for only one colour in each pixel, resulting in a colour image.

**Figure 1:**
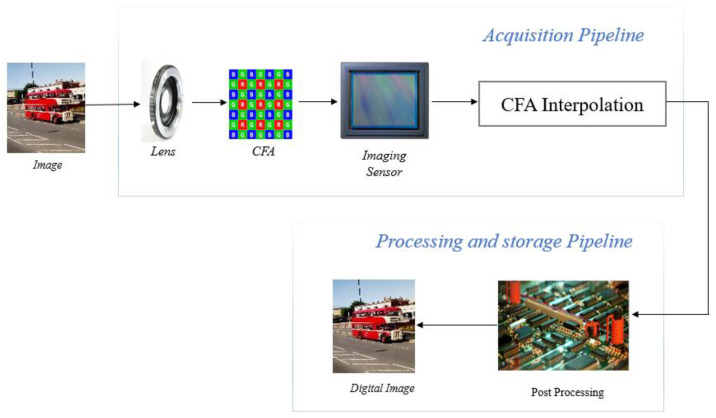
Digital camera image acquisition and processing pipeline.

The green-red-green-blue (GRGB) Bayer pattern CFA is used by most digital cameras. Red, green, and blue pixels of varying intensities make up the mosaic of colours produced by the Bayer-filtered sensor. Since each pixel can only store one of the three colours, the DIP uses a variety of interpolation (demosaicking) algorithms to create a full-colour image. Van Lanh et al. [7] opined that the cyan-yellow-green-magenta (CYGM), red-green-blue-emerald (RGBE), and cyan-magenta-yellow (CMY) patterns are further substitutes for CFA filters. In addition to interpolation, the DIP performs additional processing to create high-quality images, including noise reduction, matrix manipulation, picture sharpening, aperture correction, gamma correction, and white balancing. Figure 1 shows a block diagram of the digital camera image acquisition and its processing pipeline.

Each block of the digital camera pipeline adds distinct artefacts or patterns to the output digital image it captures. This distinct pattern is extracted and analyzed, and the information obtained is used to identify the camera type, brand, model, and more precisely the camera used to capture the image.

## 3. Image Source Camera Identification Techniques

**Figure 2:**
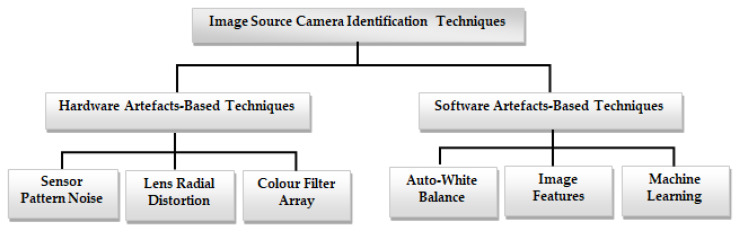
Taxonomy of image source camera identification techniques.

To help with image forensic investigations, researchers introduced different methods for image source camera identification [9,10]. This section gives a comprehensive overview of the various proposed methods for identifying the source camera of an image. This examination delves into existing methods for image source camera identification, including methods based on intrinsic hardware artifacts resulting from manufacturing imperfections, and those utilizing software-related properties. Intrinsic hardware-related flaws that can be exploited in image source camera identification include sensor pattern noise, lens radial distortion, and sensor dust, among others. Software artifact-based methodologies are used in camera fingerprint extraction using the characteristics and artifacts left by camera software, such as auto white balance approximation and colour filter array interpolation, among others. Figure 2 shows the taxonomy of image source camera identification techniques.

All the above techniques were tested using various publicly available image datasets like VISION image dataset [11], Dresden image dataset [12], and high dynamic range image dataset [13]. However, some researchers created their personal image dataset which is not placed in the public domain.

### 3.1. Sensor Pattern Noise-Based Techniques

A flaw in the manufacturing process of the image sensor chip, which creates in pixel sensitivity variation in the imaging sensor, is the source of sensor pattern noise (SPN). These pattern noises contain a distinctive quality that makes them identifiable to that camera imaging sensor. Therefore, it provides a “fingerprint” of that specific digital camera. The main component of SPN is the photo response non-uniformity (PRNU) noise. Therefore, analyzing the PRNU noise, which is measured as a unique camera fingerprint, is one of the trustworthy techniques for image source camera identification using SPN. The image still undergoes further processing stages like demosaicking, interpolation, and gamma correction after the sensing process. Even after going through all of this, the image still has bullet scratches which are not removable by the above processes.

In the paper, which has been thought of as a benchmark for image source camera identification using SPN, Lukas et al. [14], introduced a technique that uses discrete wavelet transform to decompose the original images into four sub-bands. Then it applies a Wiener denoising filter on the resulting three high-frequency wavelet subbands to denoise the image high-frequency wavelet subbands and reconstruct the image using the smoothed wavelet high-frequency sub-bands. It subtracts the resulting denoised image from the input image to compute the reference pattern noise of the image. The camera fingerprint is computed by averaging the reference pattern noise of a few images from the camera under different conditions. Then, to determine if the image comes from the reference camera, they use the normalization cross correlation between the calculated pattern noise of the injury image and the pattern noise of the camera. Even though this method appears to have the potential to increase computing complexity and cannot be used for large-scale processing, its level of reliability tends to be high. The experiments were conducted on roughly 320 images captured by nine consumer digital cameras, and the outcomes of the experiment were assessed using false acceptance rate (FAR) and false rejection rate (FRR) error rates. Even for cameras of the same model, the camera recognition is 99.8% accurate. Jaiswal and Srivastava in [15] highlighted that image scenes may highly contaminate the extracted PRNU, resulting in wrong camera identification. Therefore, they proposed a framework based on the frequency and spatial features to increase the size of the image dataset used to train and estimate the camera PRNU. The proposed framework uses discrete wavelet transform (DWT) and local binary pattern (LBP) to extract features from the images. These features are then used to train a multi-class classifier, e.g., support vector machine (SVM), linear discriminant analysis (LDA), and K-nearest neighbor (KNN). The resulting trained classifier is then used to identify the image camera source. Soobhany et al. [16] proposed another technique like [14] where they used a non-discrete wavelet transform to decompose the input image into four wavelet sub-bands. To calculate the SPN from the image, the coefficients of the resulting wavelet high-frequency sub-bands are de-noised. The image SPN signature was compared to the camera reference SPN signature to identify the image source camera. An advantage of this technique is that the non-decimated wavelet transform maintains all the details of the wavelet sub-bands during the decomposition process allowing for more information to be preserved. Again, the SPN signature can be retrieved after the first level of wavelet decomposition, as compared to the decimated approach, which requires four levels of wavelet decomposition to obtain a credible SPN. The proposed method was tested using images from ten different cameras from the Dresden image dataset. Results demonstrate that the suggested method outperforms the state-of-the-art wavelet-based image source camera identification method with relatively low computational cost. Al-Athamneh et al. [17] suggested the use of only the green component of an RGB image for PRNU extraction while using a similar method used in [14]. This is because human eyes are susceptible to green colour, and the green colour of the sensor pixel caries twice the information compared to its red and blue components. The green colour channel of the video frames was examined to create G-PRNU (green—photo response non-uniformity). The technique demonstrated a good level of reliability in identifying digital video cameras and generated superior performance compared to PRNU in identifying the source of digital videos. Images from six cameras were used to test the technique (two mobile phones and four consumer cameras). Videos, 290 in number, were recorded over the course of four months in a variety of settings. The 2-D correlation coefficient detection test was used to determine the sources of each of the 290 test videos. Their results show an average prediction accuracy of 99.15%. Akshatha et al. [18] proposed an image camera source identification technique. They used a high-order wavelet statistics (HOWS) method to remove the camera noise from the input image and extract the camera signature. To determine the originating source camera for the given image, the features were fed to support vector machine classifiers, and the results were validated using the ten-fold cross-validation technique. Images taken with different cell phone cameras were used, and the algorithm proved to be capable of accurately identifying the source camera of the provided image with 96.18% accuracy on average, irrespective of camera model or band. Georgievska et al. [19] proposed an image source camera identification method where images are clustered based on peak to correlation energy (PCE) similarity scores of their PRNU patterns. The image is first converted to grayscale. The initial estimate of the PRNU pattern is obtained using the first step total variation (FSTV) algorithm. After that zero mean and Wiener filtering steps are performed to filter out any artefacts produced by colour interpolation, on-sensor signal transfer, imaging sensor design, and JPEG compression. Then, PCE is computed as the ratio between the height of the peak and the energy of the cross correlation between two PRNU patterns. Their proposed technique uses graphics processing units (GPUs) to extract the PRNU patterns from large sets of images as well as to compute the PCE scores within a reasonable timeframe. The performance of the proposed method was evaluated using the Dresden image dataset. Their result showed this technique is highly effective.

Rodrıguez-Santos et al. [20] proposed employing Jensen–Shannon divergence (JSD) to statistically compare the PRNU-based fingerprint of each qualifying source camera against the noise residual of the disputed image for the digital camera identification technique. Zhang et al. [21] proposed an iterative algorithm tri-transfer learning (TTL) for source camera identification, this algorithm combines transfer learning with tri-training learning. The transfer learning module in TTL transfers knowledge obtained from training sets to improve identification performance. In comparison to previous methods, combining the two modules allows the framework to achieve superior efficiency and performance on mismatched camera model identification compared to other state-of-the-art techniques. Zeng et al. [22] proposed a dual tree complex wavelet transform (DTCWT)-based approach for extracting the SPN from a given image that performs better near strong edges. Symmetric boundary extension rather than periodized boundary extension was used to improve the quality of SPN as well as the picture border. Balamurugan et al. [23] proposed an image source camera identification technique, which uses an improved locally adaptive discrete cosine transform (LADCT) filter followed by a weighted averaging method to exploit the content of images carrying PRNU efficiently. LADCT is believed to perform well on images with high image-dependent noise like multiplicative noise of which PRNU is one of such. The technique divides images into blocks of fixed size in pixels that can be shifted in a single step either horizontally or vertically. A discrete cosine transform (DCT) is applied on each block, extracting its DCT coefficient, and for each of the provided blocks and over the DCT coefficients, and a threshold is applied. With the application of inverse DCT (IDCT) on the DCT coefficients, the blocks are once more reconstructed in the spatial domain. Then the average of the DCT coefficients for the same spatial domain values is used to determine the final estimation of the pixel. The weighted average provides weight to every coefficient of the blocks with the same weights, providing a greater averaging value than the simple average. The Dresden image dataset was used to evaluate the performance of the proposed technique. Their experimental results demonstrated its significant effectiveness. Qian et al. [24] introduced a source camera identification technique for web images using neural-network augmented sensor pattern noise to easily trace web images while maintaining confidentiality. Their technique includes three stages: initial device fingerprint registration, fingerprint extraction, secure connection establishment during image collection, and verification of the relationship between images and their source devices. This technique provides cutting-edge performance for dependable source identification in modern smartphone images by adding metric learning and frequency consistency into the deep network design. Their technique also offers many optimisation sub-modules to reduce fingerprint leakage while improving accuracy and efficiency. It uses two cryptographic techniques, the fuzzy extractor and zero-knowledge proof, to securely establish the correlation between registered and validated image fingerprints.

Lawgaly and Khelifi [25] proposed similar techniques that use locally adaptive DCT (LADCT) for image source camera identification. Their technique enhanced the locally adaptive DCT filter before the weighted averaging (WA) approach as in [23] to effectively exploit the content of images conveying the PRNU. The estimated colour PRNUs were concatenated for better matching because the physical PRNU is present in all colour planes. The system was thoroughly evaluated via extensive experiments on two separate image datasets considering varied image sizes, and the gain obtained with each of its components was highlighted. To produce denoised estimates of neighboring and overlapping blocks, they used a sliding block window. The local block means and the local noise variance both influence each block’s threshold. The algorithm was evaluated using images from the Dresden dataset; their results demonstrated superior performance against cutting-edge techniques. Chen and Thing [26] adopted what they called block matching and 3D filtering (BM3D) which is known as a collaborative filtering process. This proposed technique grouped similar blocks extracted from images where each group is stacked together to form 3D cylinder-like shapes. Filtering is performed on every block group. A linear transform is applied on the image before Wiener filtering. Then, the transform is inverted to reproduce all filtered blocks before the image is transformed back to its 2D form. Their results show that PRNU-based methods can provide a certain level of capability in terms of verifying the integrity of images. However, increasing the number of images utilized for PRNU pattern estimate might enhance performance but it would also make the approach less practical. 

Yaqub [27] proposed a simple scaling-based technique for image source camera identification when the questioned image is cropped from an unidentified source or when it is full resolution. The technique presents a simple, effective, and efficient approach for image source camera identification based on a hierarchy of scaled camera fingerprints. Lower levels of the hierarchy, which contain scaled-down fingerprints, allow for the elimination of many candidate cameras, which reduces computation time. Test results show that the technique while being applicable to full-resolution and cropped query images, leads to significantly less computation. A test with 500 cameras showed that for non-cropped images, the technique has 55 times less run time overhead than the conventional full-resolution correlation, while for cropped images, the overhead is decreased by a factor of 13.35. Kulkarni and Mane [28] proposed a hybrid system made up of the best results as a method for extracting sensor noise that uses gradient-based operators and Laplacian operators to generate a third image while also revealing the noise and edges present in it. To obtain the noise present in the image, a threshold is applied to remove the edges. 

**Figure 3:**
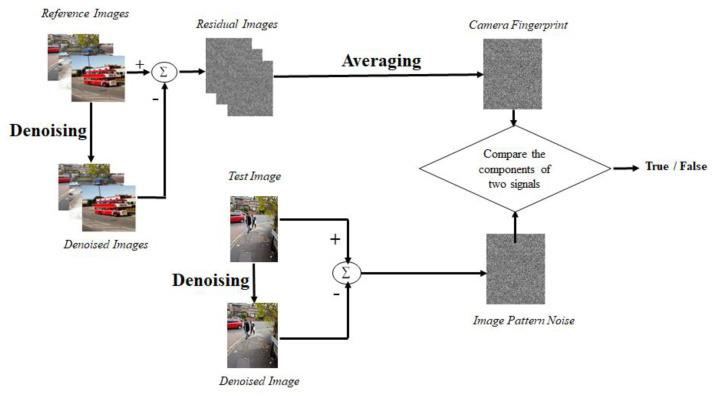
SPN processing pipeline using large components of sensor pattern noise.

The gray level co-occurrence matrix (GLCM) in the feature extraction module is then given this noisy image. Based on its qualities, homogeneity, contrast, correlation, and entropy are used to extract numerous features. To obtain an exact match, the SPN is retrieved from the GLCM features and used for matching with the test set. Results are improved by the hybrid method that combines GLCM feature extraction with SPN extraction. Using Dresden image dataset, the technique’s accuracy is found to be, on average, 97.59%, which is quite high. Figure 3 shows the flow chart for source camera identification using large components of sensor pattern noise.

The effect of wavelet transform on the performance of the conventional wavelet-based image camera source identification technique was reported in [29]. The authors used plane images from the VISION image dataset captured using eleven different camera brands to generate the experimental results. They reported that the conventional wavelet-based technique achieves its highest performance when it uses a sym2 wavelet.

### 3.2. Intrinsic Lens Radial Distortion

In a camera, a lens is a device that directs light toward a fixed focal point. The symmetric distortion caused by flaws in the lens’s curvature during the grinding process is known as radial lens distortion. Most image devices, as mentioned by Choi et al. in [30], have lenses with spherical surfaces; their intrinsic radial distortions act as a distinctive fingerprint for recognizing source cameras. In this paper, the authors introduced two kinds of features based on pixel intensities and distortion measurements, which enable measurement of the radial distortions causing a straight line to become curved in the images. Performing four different sets of experiments, lens radial distortion in image categorization is used in the initial set of tests as a feasibility test. The second set of tests demonstrates that the technique outperforms those that solely use image intensities in terms of accuracy by a statistically significant margin. The next series of tests examines how the suggested features work when more cameras and testing images are considered. The fourth set of tests examines how the focal length of zoom lenses affects error rates. The SVM classifier included in the LibSVM package was utilized in the tests and the average accuracy obtained was 91.5% associated with the confusion matrix as assessment criterion. 

**Figure 4:**
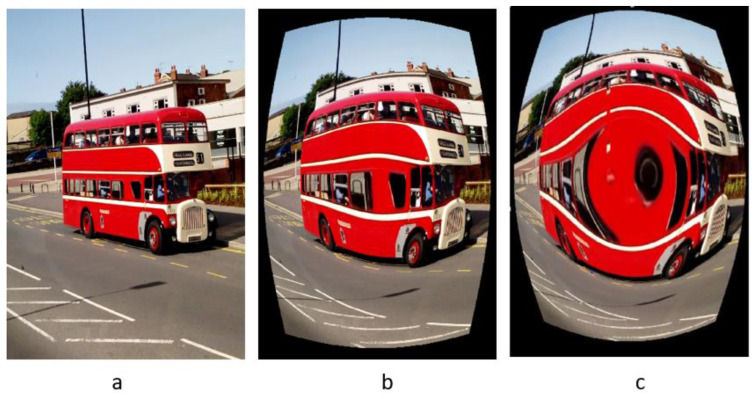
Sample of lens radial distortion: (**a**) original, (**b**) barrel distortion, (**c**) pincushion distortion.

Bernacki [5] reported a digital camera identification technique using a real-time image processing system based on the investigation of vignetting and distortion flaws. The technique eliminates the need for a wavelet-based denoising filter or the creation of camera fingerprints, both of which have a significant impact on the image processing speed. Instead, the technique separates the red colour band from the input image and filters it using a median filter. After that, the absolute difference between the red colour channel and its median filtered version is computed. The size of the picture sections to be examined at the four corners is determined, and the mean value of the pixel intensities is computed to provide the value that will be utilized as the camera signature. Their findings suggest that vignetting defect analysis can be used to identify camera brands with less computational effort. This technique calculates the distortion parameter k for a collection of images taken with various cameras to see if there are any patterns that could be used to identify an individual camera using this model p_u_ = p_d_ (1 + kr^2^). Brand identification accuracy on smartphones and the Dresden image dataset is 72% and 52%, respectively. As a result, the performance accuracy is less than the algorithm presented in [13], but the vignetting-CT algorithm outperforms it in terms of speed. Figure 4 displays an example of lens radial distortion highlighting the original scene, barrel, and pincushion distortions. 

### 3.3. Colour Filter Array Interpolation

Colour filter array (CFA) is a demosaicing method used in digital cameras. It is also known as a colour reconstruction method, which is used to reconstruct a digital colour image from the colour samples generated by an image sensor overlaid with a CFA. This demosaicing information can be extracted and used as a camera fingerprint.

**Figure 5:**
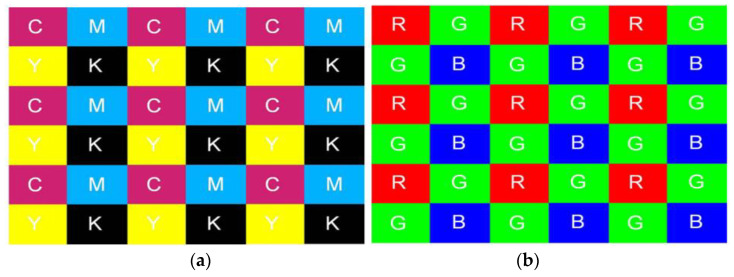
CFA pattern using (**a**) CMYK values and (**b**) RGB values [31].

To discern the correlation structure present in each color band for image classification purposes, Bayram et al. [31] investigated the CFA interpolation procedure. The underlying assumption is that each device manufacturer’s interpolation algorithm and CFA filter pattern design exhibit distinct uniqueness, leading to discernible correlation structures in captured images. Utilizing the iterative expectation maximization (EM) algorithm, two distinct sets of features are derived for classification: the interpolation coefficients derived from the images and the peak locations and magnitudes within the frequency spectrum of the probability maps. Two camera models: Sony DSC-P51 and Nikon E-2100 with a resolution of two megapixels are used in the dataset. Using the confusion matrix for assessment the classification accuracy is 95.71% for two separate cameras when using a 5 × 5 interpolation kernel, however, it decreases to 83.33% when three cameras are compared. It ought to have been investigated how this technique affected the categorization accuracy with a larger number of cameras. The technique has not been tested with identical model cameras, but failure could be anticipated because identical model cameras often utilize the same CFA filter pattern and interpolation algorithm. Consequently, this technique may not perform well where compressed images are involved. Figure 5 shows the Bayram RGB interpolation values.

Lia and Lin [32] introduced an algorithm that employs an interpolation of images to determine image characteristic values with a support vector machine (SVM) to lower the required processing power and attain a high true positive. This algorithm uses the colour interpolation methods, which includes bilinear interpolation, adaptive colour plane interpolation, effective colour interpolation and highly effective iterative demosaicking. Cameras of various brands and models were employed to conduct classification in the study and the results of their study showed that this method had a good identification rate, with a recognition rate of up to 90% only when a wave filter was additionally introduced. Chen and Stamm [33] proposed a camera brand identification technique. Their method first re-samples colour components of the input image in relation to a predetermined CFA pattern, where M different baseline demosaicing algorithms are applied to demosaic missing colour components in the input image. It then subtracts each resulting re-demosaic image from the input image generating M demosaic residual images. The resulting demosaic residual images are considered as a set of co-occurrence matrices using K different geometric patterns. It then uses the multi-class ensemble classification method to extract the camera brand signature. They used relative error reduction (RER) criteria to measure the performance of their technique. They reported a performance of 98% in terms of accuracy for camera model identification using images from the Dresden image dataset.

### 3.4. Machine Learning

Machine learning technology is being steadily incorporated into the field of image forensics with the evolution of artificial intelligence and the development of available image datasets. Moreover, machine learning technology can extract most appropriate features from a range of training datasets, suppressing the drawbacks of features that were generated artificially. Ahmed et al. [34] introduced a deep convolutional neural network for a source camera identification algorithm that employs a max pooling layer, three convolutional layers with batch normalization, a rectified linear unit as an activation function, one fully connected layer, a drop out layer, and a classification layer as its first few layers. Significantly lower training images are used to train the network to determine the source of an image, and the noise pattern of the images is determined using the algorithm reported in [14]. False positive rate (FPR) and false negative rate (FNR) are computed to assess the performance of both approaches using image datasets taken from eleven different cameras using the same set of training and test images with dimensions 128 × 128 and 256 × 256 for both approaches. Their research demonstrates that the PRNU-based technique is more effective than the convolutional neural networks (CNN) based approach. Marra et al. in [35] and Freire-Obregón et al. in [36] used CNN which is a subset of machine learning and is composed of multiple layers, with each layer containing a set of high-pass filters applied across the input image, for camera source identification. The convolutional process is used for the automatic extraction of features from the data and the subsequent learning from these extracted features. Their result shows an accuracy level of 98.1% for camera model identification and 91.1% accuracy for individual camera identification. Kirchner and Johnson [37] proposed a technique that uses CNN to train and estimate the camera signature and then compute the noise residual from the test images and uses the maximum likelihood fingerprint estimator (MLE) to estimate the fingerprint of the test images. Using VISION image dataset and Dresden image dataset, the study proved that using a deep learning technique can result in a more appropriate extractor, which leads to better source attribution as it achieves the best results using a certain set of criteria for each potential camera fingerprint.

Ding et al. [38] reported an algorithm which has one pre-processing module, one feature extractor, and one hierarchical multi-task learning method. The pre-processing module uses domain information for the deep learning method of camera identification. By distributing the knowledge across all the tasks, a hierarchical multi-task learning approach contributes more supervised information to the classification problem. The ResNet can combine low-, mid-, and high-level features and reuse earlier features through shortcut connections. The input image is first transferred to the pre-processing module to generate intermediate features; these features are then sent into the deep network, which is used to identify cameras. The convolutional layer is denoted as “Conv.” Resnet3_1, Resnet4_1, and Resnet5_1 execute down-sampling with a stride of 2. The functions classify 1, classify 2, and classify 3 are used to distinguish between camera brands, modes, and devices. Using original and altered images for assessment, the framework was assessed for brand, model, and device-level identification and the result shows that the technique is robust and reliable. The findings showed a significant improvement in the accuracy of mobile phone device identification to an average of 84.3%, better than consumer-level camera device identification. The result experimented on the Dresden dataset and the cell phone dataset using the t-SNE analysis. 

Liu et al. [39] reported a proficient source camera identification method based on convolutional neural networks. Their method has three essential components: patch selection based on multiple criteria, fine-grained multiscale residual prediction, and a modified visual geometry group (VGG) identification method. Authors argued that the conventional source camera identification’s performance is influenced by image content and falls short of meeting the demands of real-world applications, particularly for small image patches. The proposal advocated the division of all training and test images into 64 × 64 nonoverlapping patches with underlying distributions representative of all training and testing patches. These representative patches obtained by the patch selection module are used as training data to supervise the learning of subsequent residual prediction and classification throughout the training phase. All patches in test images are recognized for final performance evaluation after the parameters have been trained. This boosts robustness while lowering training costs, and representative patches are chosen based on a variety of parameters to increase training data diversity. At the brand, model, and instance levels, a modified VGG network was presented for source camera detection. According to them, this approach performed admirably in terms of both identification accuracy and computational efficiency. Using the Dresden dataset and the classification confusion matrix visualization of the 18 camera models, the classification accuracy of most camera models is higher than 97%. Huang et al. [40] reported a convolutional neural network-based technique for determining the source camera of digital images. The technique depends on constructing a new network that includes an input layer, three convolutional layers with max pooling and normalization, two fully connected layers, and the Softmax classifier. To reduce the size of sample images from the targeted camera that the network needs to use as training data, the original images are cropped into small-sized patches that the network is meant to assess. To identify the source camera, a local-to-global technique is also implemented that respects the principle of majority voting among the image patches. Using images from the Dresden dataset and confusion matrix as assessment criteria the technique reached an accuracy of up to 99.8%, according to test results. Timmerman [41] proposed an improved restricted convolutional layer that can handle colour inputs and can handle inputs with three colour channels. Colour inputs require three kernels as opposed to one kernel for grayscale inputs. While [40] used images in their technique, ref. [41] used video frames to extract and source the camera using SPN. The method was created to categorize distinct video frames, which are then combined by a majority vote to identify the originating camera. The benchmark VISION data set, which contains 1539 videos from 28 distinct cameras, was used to evaluate the technique. The tests revealed that the method was resistant to the compression methods used by YouTube and WhatsApp and still managed to reach up to 93.1% accuracy. Bondi et al. in [42] and Kang et al. in [43] proposed different CNN based techniques for source camera identification, which generate a high level of accuracy. For protection against malicious adversarial attacks on source camera identification, Hui et al. in [44] proposed a defense mechanism to counter adversarial attacks in the source camera identification problem. This involves analyzing the image acquisition process, refining the source camera identification problem and its adversarial attacks, modeling feature extraction, and deriving a defense objective based on information monotonicity to suppress adversarial noise amplification during mapping. Additionally, local smooth mapping was used to reduce mapping oscillation. To address the training cost and migration challenges of existing solutions like adversarial training, they implemented a two-phase migratable pre-defense network. Their result showed that the source camera identification model combined with a pre-defense network maintained high identification accuracy and improved adversarial robustness compared to other defense models. 

In [45], a unified architectural representation of source camera identification powered by a deep neural network was introduced. The proposed method extracts the residue noise from each input image by first denoising the input image using a U-net and then subtracting it from the input image. The resulting image residual noises are then encoded into an embedding using a feature modulator, where they are conditioned on the triplet loss function to minimize the distance between images from the same camera and maximizes the distance between different images. Finally, the one-shot method is used to determine the camera source of the image. Their results showed that their method achieved 97.59% and a 97.01% in terms of F-score accuracy, respectively. In [46], an adaptive dual-branch fusion residual network based on the SE-BRB module to improve network performance for image camera source identification was presented. The authors claimed that the network is relatively simple in terms of complexity and can be used for small size source identification. They reported a performance of 99.33% in terms of accuracy on images of the Dresden dataset.

### 3.5. Auto-White Balance (AWB) Approximation

White balance balances the colour temperature of an image. To return the colour temperature to neutral, it adds the opposing colour to the image. After white balancing an image, whites should appear white rather than orange or blue. The distinctive attribute of AWB known as idempotence ensures that there will not be any difference in the results if the same AWB method is used twice or more. Deng et al. [47] proposed a technique for identifying source cameras using auto-white balance approximation (AWB). This technique involved colour adjustment to make the image look natural by removing the colour cast. The original image is first resampled and various auto-white balance (AWB) is applied to approximate the method that may be used inside the camera. Image features are then extracted, and feature vectors are selected using sequential backward feature selection and the prediction of the source camera is achieved using a support vector machine (SVM) classifier. The study using the Dresden image dataset, which consisted of around 29 cameras and devices with 17 models and 8 brands, produced the following predictions. The average prediction for cameras of various brands was 99.26%, while for cameras of various models, the average prediction was 98.61%. The average prediction for cameras of the same model was 98.57%. Arathy et al. [48] applied the same technique as in [42] using AWB approximation for source camera identification. However, while [42] used SVM classifier for prediction, [43] compared the performance of SVM and neural network (NN) classifiers. Using many images from various camera models and sub-models and using receiver operating characteristics (ROC) as an assessment criterion, their result showed that SVM prediction had an accuracy of 99.67% whereas NN prediction had a lower accuracy of only 92.92%.

### 3.6. Image Features-Based Techniques

An image feature is a piece of data about an image’s content that is used in computer vision and image processing by applying data mining techniques. It often pertains to whether a certain section of the image possesses unique characteristics. Features in an image can be particular elements like points, edges, or objects. To identify the camera sources of the images, these features were trained and classified. Tsai and Wu [49] proposed an image camera source identification technique that used a total of 33 features to identify the source cameras. These features were grouped into three categories: colour features, image quality features, and wavelet domain features. Features under colour features include average pixel value, RGB pairs correlation, neighbor distribution center of mass and RGB pairs energy ratio. Features under image quality include mean square error, MSE, mean absolute error, and Minkowski difference for pixel difference-based; structural content, normalized cross correlation, and Czekonowski correlation for correlation-based; spectral magnitude error, spectral phase error, spectral phase-magnitude error, block spectral magnitude error, block spectral phase error, and block spectral phase-magnitude error for spectral-based. The means for each of the three resulting high frequency sub-band coefficients of each image color band was calculated and used as features to determine the image camera source. LibSVM was the classifier used in this paper which aids in categorizing incoming data and assessing the accuracy rate. The method discovered that the feature-based approach significantly outperforms other camera brand identification methods.

Xu et al. [50] proposed an algorithm that uses image texture attributes that are taken from the carefully chosen colour model and colour channel for source camera identification. With this technique, the local phase quantization (LPQ) features are extracted from the original images and the residual noise images, whilst the LBP features are extracted from the original images and the residual noise images, respectively. In the HSV colour space, the H and V colour channels are used to extract the LBP and LPQ features. After that, the combined LBP and LPQ features are fed into the multi-class LibSVM classifier for source camera prediction. The technique has satisfactory detection accuracy and resilience, according to its result, distinguishing camera brands and models, camera models from the same brand of cameras, and camera individuals from the same model and brand of cameras. Three functions are envisaged to be accomplished by the proposed method: distinguishing camera brands and models; models from the same brand of cameras; and individual cameras from the same model and brand of cameras. Using the Dresden image dataset, the results show that the performance of this technique is satisfactory when compared with the state-of-the-art techniques. A summary of different existing image camera source identification methods, the datasets, and the assessment criteria they used with the accuracy achieved is tabulated in Table 1.

An overview of some publicly available image datasets for camera identification is presented in Table 2. VISION image dataset was captured using 35 portable devices of 11 major brands, containing 34,427 images and 1914 videos, both in native and social media formats. Images are made up of plain and textured images and all images are in JEPG format [11]. Dresden image dataset contains over 14,000 images that are made up of various indoor and outdoor scenes, captured using 73 digital cameras of 25 different models. All images are in JEPG format [12]. High dynamic range image dataset contains more than 5000 images captured using 23 different mobile devices of 7 major brands. Images are in JEPG format and made up of plain and textured images [13]. Forchheim image database consists of more than 23,000 images of 143 scenes by 27 smartphone cameras. Each image is provided in six different qualities: the original camera-native version, and five copies from social networks. All the images are in JPG format [51].

## 4. Conclusions

This paper explored the performance of existing source camera identification methods for both intrinsic hardware artifacts-based algorithms like sensor pattern noise (SPN), lens optical distortion, and software artifacts-based methods like CFA, auto white balancing, and learning-based techniques. Findings reveal that while SPN and lens radial distortion were able to achieve individual camera identification, CFA, auto-white balancing, and machine learning techniques were only able to achieve camera model identification. Sensor pattern noise gives the best performance result in terms of accuracy in source camera identification at a camera level identification of 99.8%. However, this approach is computationally more expensive than others. It is followed by lens optical distortion-, auto-white balancing- and deep learning-, and colour filter array-based methods in terms of their computational costs, which achieve 97.59%, 98%, and 95.71%, respectively. Notably, the last three techniques exhibited proficient performance but were limited to camera model level identification, lacking the capability to distinguish between sets of images from different cameras of the same model.

VISION and Dresden image datasets have been widely used by the researchers; however, some used author-generated image datasets and using assessment criteria like FAR/FRR Error Rate, ROC, TPR/FPR, FPR and FNR, confusion matrix, relative error reduction (RER), t-SNE analysis, and accuracy and error rate. It is not believed that the image dataset used influenced the result anywhere but using publicly available tested image datasets like VISION and Dresden image datasets will help standardize the findings.

## 5. Recommendation for Future Work

Many wavelet-based image camera source identification methods have been reported in the literature. These techniques use different wavelets; however, there is no research to determine the effect of the used type of the wavelet on the performance of these techniques. In addition, researchers have used various publicly available image/video datasets to assess the performance of their methods. There is need for a comprehensive image/video dataset covering significantly more devices. Simulation results for different image camera source identification methods show that the performance of these methods depends on image contents and is significantly deteriorated when large areas of the image contents are textures. Therefore, there is a demand for techniques to overcome these limitations. Finally, with the increase in the volume of social media images, the demand for fast and efficient techniques are also increasing. 

## Figures and Tables

**Table 1:** Summary of the relevant bibliography for image camera source identification techniques. (Sensor Pattern Noise (SPN), Lens Radial Distortion (LRD), Colour Filter Array (CFA), Machin Learning (ML), Automatic White Balancing (AWB), Image Features (IF), and Relative Error Reduction).

Ref.	Image Dataset	Used Method	Assessment Criteria	Identification Level	Accuracy
[5]	Dresden	SPN	TPR/FNR	Individual Camera	72% with low computational time
[14]	Authors	SPN	FAR/FRR Error Rate	Individual Camera	99.8%
[16]	Dresden	LRD	ROC	Individual Camera	Very high
[17]	Authors	SPN	FAR/FRR	Individual Camera	99.15%
[18]	Authors	SPN	FAR/FRR	Individual Camera	96.18%
[19]	Dresden	SPN	TPR/FPR	Individual Camera	89%
[24]	Dresden	SPN	ROC	Individual Camera	Excellent
[26]	Dresden	SPN	ROC	Individual Camera	Superior Performance
[30]	Dresden	LRD	FPR and FNR	Individual Camera	97.59%
[31]	Authors	CFA	Confusion Matrix	Camera Model	91.5%
[32]	Authors	CFA	Confusion Matrix	Camera Model	95.71%
[33]	Authors	CFA	ROC	Camera Model	90%
[34]	Dresden	ML	RER	Camera Model	98%
[39]	Dresden	ML	t-SNE analysis	Camera Model	83.3%
[40]	Dresden	ML	Confusion Matrix	Camera Model	97%
[41]	Vision	SPN&ML	Confusion Matrix	Individual Camera	93.1%
[42]	Vision	ML	Relative Error Reduction	Camera Model	96.8%
[47]	Dresden	AWB	ROC	Camera Model	98%
[48]	Authors	AWB	ROC	Camera Model	SVM 96.67% NN 92.92%
[50]	Dresden	IF	Confusion Matrix	Camera Model	97.75%

**Table 2:** Overview of publicly available and widely image dataset for source camera identification.

Dataset Name		Vision [10]	Dresden [11]	HDR [12]	Forchheim [46]
No. of Images		34,427	14,000	5000	23,000
No. of Devices		35	73	25	27
No. of Device Models		11	24	7	25
Device Type	Camera Phone	20	50	15	27
	Digital SLR Camera	15	23	10	0
Image Format		JEPG			
Scenes		Indoor and Outdoor			
Visual Content		Plain and Textured Images			
Availability		Publicly Available			

## Data Availability

Not applicable.
